# Effects of Hemp Seed on the Production, Fatty Acid Profile, and Antioxidant Capacity of Milk from Goats Fed Hay or a Mixed Shrubs–Grass Rangeland

**DOI:** 10.3390/ani13223435

**Published:** 2023-11-07

**Authors:** Daniel Mierlita, Stefania Mierlita, Danut Ioan Struti, Olimpia Smaranda Mintas

**Affiliations:** 1 Department of Animal Nutrition, Faculty of Environmental Protection, University of Oradea, 1 University St., 410087 Oradea, Romania; dadi.mierlita@yahoo.com (D.M.); olimpia.mintas@uoradea.ro (O.S.M.); 2 Department of Accounting and Audit, Faculty of Economics and Business Administration, Babes-Bolyai University, 58-60 Teodor Mihali St., 400372 Cluj-Napoca, Romania; stefania.mierlita@econ.ubbcluj.ro; 3 Department of Technological Science, Faculty of Animal Science and Biotechnologies, University of Agricultural Sciences and Veterinary Medicine, 3-5 Manastur St., 400372 Cluj-Napoca, Romania

**Keywords:** goat milk, hemp seeds, fatty acid composition, health lipid indices, fat-soluble vitamins, antioxidants activity

## Abstract

**Simple Summary:**

There is increasing consumer demand for goat’s milk because it contains more nutrients and biologically active compounds than cow’s milk. However, the nutrition of goats can influence the composition of the milk, which can affect its functional activity. This research evaluates the effect of dietary inclusion of hemp seeds in goats fed with hay or mixed shrubs–grass rangeland, on the yield, fatty acid profile (FA), and antioxidant capacity of milk, with the goal to increase the content of its beneficial ingredients. The results obtained led to the conclusion that grazing goats on mixed shrubs–grass rangeland (SG) and dietary supplementation with hemp seed (Hs) is an effective strategy for increasing the milk’s fat content and improving its quality in terms of FA profile, lipophilic antioxidant content, and total antioxidant capacity (TAC), making goat’s milk a food with high added value that is able to provide benefits for human health.

**Abstract:**

The research objective was to evaluate the effect of dietary incorporation of hemp seeds in goats fed with hay or mixed shrubs–grass rangeland on the production, FA profile and health-related lipid indices, antioxidant content and total antioxidant capacity of milk, with the purpose to increase the content of beneficial ingredients in milk and to improve its functional activity. Forty indigenous Carpathian goats were allocated into two groups according to the type of basic forage in their diet: hay (H) or mixed shrubs–grass rangeland (SG); each of them was further divided into two subgroups according to the presence of Hs in the concentrate mixture (250 g/kg) or not. Milk production was determined, and milk samples were collected and analyzed for fat, protein, lactose, and cholesterol content, as well as FA profile, lipophilic antioxidant content (α-tocopherol and retinol), and milk TAC. SG goats gave less milk (*p* < 0.01) but with higher milk fat (*p* < 0.001) and lower cholesterol content (*p* < 0.01) than H goats, while milk protein and lactose contents were not affected. Supplementing the diet with Hs caused a significant increase in milk production (*p* < 0.05) and milk fat content (*p* < 0.001) and a decrease in cholesterol content (*p* < 0.05). Grazing compared to indoor feeding but also supplementing the diet with Hs had the effect of decreasing the proportion of SFAs and increasing the concentrations of polyunsaturated FA (PUFA) in goat milk fat (*p* < 0.01). Fats in the milk of SG goats compared to H, but also in those supplemented with Hs, showed significantly higher proportions of vaccenic acid (VA), rumenic acid (CLA c-9,t-11) and omega-3 FA (α-linolenic acid (ALA), eicosapentaenoic acid (EPA), and docosapentaenoic acid (DPA)) which are considered healthy for consumers. The feeding system based on SG and the diet supplementation with Hs ensured the best nutritional and functional quality of milk, confirmed by the FA profile, antioxidant content, and by the value of health-related lipid indices (n-6/n-3 FA ratio and hypo-/hypercholesterolemia, atherogenic index (AI), thrombogenic index (TI), and health promotion index (HPI)). The results of our work will be useful for the development of optimal nutritional strategies that improve the FA profile and the antioxidants content in goat milk, with beneficial effects on human health.

## 1. Introduction

The increased consumer interest in goat milk is due to its improved fatty acid profile compare with cow’s milk, but also due to its lower cholesterol content than other types of milk [1]. The more favorable FA composition of goat milk (such as higher omega-3 FA content and lower atherogenic FA, especially palmitic acid content) contributes to reduced human health problems [2].

Relevant studies have shown that goat milk contains bioactive compounds such as VA (C18:1 t-11), RA (CLA c-9,t-11), and n-3 FA (ALA, EPA, and DPA), which have beneficial effects on human health and the cardiovascular system and are important in cancer prevention [3]. In addition, fat in milk is a good source of vitamins (all-trans-retinol and α-tocopherol), which possess increased antioxidant activity [4]. The development of nutritional strategies to improve the FA profile of fats in goat milk is therefore essential to reduce the content of 12:0, 14:0, and 16:0 in milk due to their hypercholesterolemic potential and to increase the beneficial FA content for human health.

Numerous studies have highlighted the positive effect of pastures on the nutritional quality of goat milk, especially the content of n-3 FA and CLA (conjugated linoleic acid) [5]. In the submontane and mountain regions of Romania, milk is produced mainly by goats and sheep, which can efficiently exploit pastures located on sloping and rugged terrain, such that pastures are their main resources of feed. In the hot and dry summer months (the period from June to August, in Romania’s conditions), these resources can no longer meet the nutritional requirements of the goats due to the decrease in the productivity and quality of the pastures. The decrease in the quality of pastured plants and the negative energy balance that occurs due to the decrease in the digestibility of nutrients and the reduced dry matter (DM) intake, in addition to the decrease in milk production, also causes a decrease in the content of FAs that are beneficial for human health (VA, CLA c-9,t-11, and n-3 FA) from milk and increases the concentration of saturated FA with an atherogenic effect [6,7]. Thus, breeders have developed several goat-feeding strategies to support milk production, such as the exploitation of mixed shrubs–grass rangeland [8]. During the grazing period, goats can cover 50–80% of their feed needs by consuming different species of shrubs and bushes [7]; goats can efficiently utilize woody pastures, which are rich in cell walls and polyphenols (especially tannins), compared to cows or sheep. In addition, in the spring, when the conditions are favorable for the growth of pasture plants, farmers take advantage and build up reserves by harvesting and drying the grasses from the pasture, obtaining a hay consisting of a wide variety of grass species, traditionally called “pasture hay”. In winter and during the summer dry period, pasture hay is a staple forage for small ruminants. However, few studies are available that provide information on the effects of adopting a goat-feeding strategy during the dry summer months (the use of pastures rich in shrubs and bushes vs. feeding indoors with pasture hay) on milk production, FA composition, and the total antioxidant capacity of goat milk.

Goats prefer broad-leaved plants and often consume shrubs and bushes rather than grasses because they have a higher nutritional value [9]. For example, the protein content of acacia (*Rubinia pseudacacia*) is 221 g/kg DM, and that of hornbeam (*Carpenus betulus*) is 214 g/kg DM, both of which are higher than the protein content of alfalfa hay [7]. However, shrubs and bushes contain a number of anti-nutritional factors such as tannins, oxalates, and saponins that limit the use of nutrients [8]. On the other hand, tannins in small amounts increase the absorption of nutrients, reduce methanogenesis, and increase the content of CLA in milk [10]. In addition, mixed pastures covered with herbaceous species, shrubs, and bushes provide higher concentrations of healthy FA (VA; CLA c-9,t-11; n-3 FA) and lower proportions of hypercholesterolemic FA (12:0, 14:0, and 16:0) in goat milk fat compared to pastures consisting only of herbaceous species [7].

In addition to their high protein and fat content (25.7% and 31.6% of DM, respectively), hemp seeds (Hs) are rich in PUFA, mainly LA (56.1% of total FAs) and ALA (22.4% of total FAs) [11]. In addition, Hs contain tocopherols (α-tocopherol—4.16 mg/100 g DM), which increase the oxidative stability of sheep’s milk [11]. However, a few studies reported the use of hempseed fats in the diet of small dairy ruminants [11,12,13,14].

Previous studies have shown that supplementing goats’ diet with hemp seed oil (93 g oil/day) [12], with linseed oil (130 g oil/day) [15], or with sunflower oil (130 g oil/day) [15] did not significantly influence the concentration of C18:2 n-6 and C18:3 n-3 in milk, due to the high amount of oil in feed which affected the ruminal microbial populations but also due to the fact that fatty acids were supplied in the diet as a free form (oil) and thus were very vulnerable to biohydrogenation. In addition, the studies carried out by Mierlita [11,13] demonstrated that supplementing the diet of lactating sheep with hemp seeds in the amount of 175–180 g/sheep/day (52–57 g oil/day) led to a significant increase in the concentration of C18:3 n-3 and CLA c-9,t-11 in milk. Similar results were obtained by introducing flax seeds or hemp seeds in the goats’ diet at a level of 8.5% (on DM basis) [14]. Therefore, in the present study, hemp seeds were used in goats feed as a source of PUFA, at a level of 200 g/goat/day (66 g oil/goat/day).

Increasing the concentration of PUFA in milk intensifies the oxidation processes that can lead to a decrease in the nutritional and sensory quality of milk. Thus, it is necessary that the increase in the content of PUFA in milk is accompanied by an increase in the content of antioxidants (for example, α-tocopherol, β-carotene, retinol) to reduce their oxidation [16].

To our knowledge, there are few studies available in the literature, but also heterogeneous results, about the effect of hemp seeds on the FA profile of milk fat and the total antioxidant capacity (TAC) of milk. Also, no studies have been reported thus far that quantify the direct interaction of mixed shrubs–grass rangeland and hemp seed ration supplementation on the production, FA composition, and TAC of milk.

Therefore, the research objective was to evaluate the effect of dietary inclusion of hemp seeds in goats fed with hay or mixed shrubs–grass rangeland on the milk production and composition, FA profile, fat-soluble vitamin concentrations, and total antioxidant capacity (TAC) of milk. The main goal of the study was to obtain milk enriched in bioactive compounds by the use of hemp seeds in the goats’ feed as a source of omega-3 FA and natural antioxidants, with a role in supporting and promoting human health.

## 2. Materials and Methods

The experimental procedures and animal care conditions were in accordance with European regulations regarding the use of animals in research [17].

### 2.1. Goats and Dietary Treatments

The study was conducted on a commercial goat farm in the submontane area of the Carpathian Mountains (Bihor, Romania; geographic coordinates: 46,037′42.6″ N 22,023′34.1″ E). Carpathian goats, a native breed that represents over 80% of the goats reared in Romania, were used.

Forty goats were involved in this study, which were balanced according to parity (2nd parity), stage of lactation (days in milk: 86 ± 11.62 d), body characteristics (37.8 ± 3, 1 kg at the beginning of the experiment), and milk production (1.38 ± 0.18 kg/d at the beginning of the experiment). The goats were assigned into two groups according to the type of basic forage in the diet: hay (H) or mixed shrubs–grass rangeland (SG); each of them divided into two subgroups according to the presence of hemp seeds (Hs) in the mixture of concentrated (250 g/kg) or not. Thus, from the combination of two types of forage (H (hay) and SG (mixed shrubs–grass rangeland)) and two levels of dietary supplementation with ground Hs (0 vs. 200 g Hs/goat/day), four treatment were formed: H-C: hay diet and standard concentrate without Hs; H-Hs: hay diet concentrated with Hs; SG-C: mixed shrubs–grass rangeland and standard concentrate without Hs; and SG-Hs: mixed shrubs–grass rangeland and concentrated with Hs.

In the two hay treatments (H), the goats in each group were kept indoors in separate pens, while the groups of goats in each pasture treatment grazed together, with the concentrate mixture being administered separately depending on the treatment (SG-C and SG-Hs, respectively) during milking.

Goats in group H received pasture hay ad libitum twice a day after milking, and alfalfa hay was offered at a dose of 500 g/goat/day after morning milking. The goats in the SG group between the morning milking (06:00) and the evening (18:00) grazed traditionally extensively (led by shepherds), and the grazing route of the herd was planned to take into account the availability of forage, with the goats having the opportunity to choose high-quality feed. Overnight, the SG goats were kept in a barn.

The pasture used for grazing goats in the SG group was covered mainly by shrubs and bushes (75–85%) with a height of 0.4–2.6 m; the most abundant species were *Rubinia pseudoacacia*, *Carpenus betulus*, *Prunus spinosa*, and *Rosa canina*. Herbaceous species were also present in these pastures, with the most common species being *Agrostis capillaris*, *Festuca arundinacea*, *Festuca rubra*, and *Phleum pratense*.

All goats received 800 g of concentrate/head, twice a day in equal amounts during milking, without refusal. The concentrates were isoenergetics and isonitrogenous. In the case of the H-Hs and SG-Hs groups, ground hemp seeds were included in the concentrate mixture at a rate of 25%. Thus, the goats in the H-Hs and SG-Hs groups received a daily supplement of 200 g of Hs, which ensured a fat intake of approximately 66 g/day/goat. The concentrate supplement represented approximately 30% of the total DM of the diet, and the hemp seeds represented, on average, 9% of the total DM of the diet.

The experimentation period lasted thirteen weeks, from the beginning of June to the end of August 2022. The first three weeks were used as a period for the adaptation to the dietary treatments.

### 2.2. Sample Collection

In Weeks 2, 4, 6, 8, and 10, forage samples (shrubs, grass, pasture hay, alfalfa hay, and concentrate) were collected to determine the chemical composition and FA content. Grass samples were taken from four 10 m × 25 m plots randomly distributed across the pasture (approximately 14 ha), with three subplots (0.5 m^2^) per plot, resulting in 12 subplots in total. Woody pastures were sampled by manually pulling plant parts observed to be grazed by goats [18]. Hay and concentrate samples were collected at the same time as for pasture. Each sample was taken in three replicates, which were then pooled into a single sample for each week. From these, 0.3 kg samples of feed were selected for laboratory analyses, packed in plastic bags, frozen, and stored at −20 °C.

During the experimentation period (10 weeks), weekly, bulk milk samples were taken from each group of goats in 4 × 50 mL plastic tubes. To one of the samples, we added a tablet of Bronopol (D&F Inc., Pharr, TX, USA), and it was analyzed for proximate composition. Milk samples used for FA profile analysis were frozen at −20 °C until analysis. Milk samples used for antioxidant TAC analysis of milk were wrapped in aluminum foil to protect them from light. Raw milk samples were frozen at −80 °C until analysis. All samples were transported to the laboratory in ice boxes to avoid any change in their quality.

Pasteurized milk was obtained by heating at a temperature of 63 °C for 30 min [19]. Pasteurized milk samples were stored for 4 days in a household refrigerator at 2 °C, after which they were analyzed for α-tocopherol and retinol content and for TAC.

### 2.3. Chemical Analyses

#### 2.3.1. Feed and Milk Analysis for Proximate Composition

The feed samples were analyzed for the content of dry matter (DM) using the gravimetric method and crude protein (CP) content using the Kjeldahl method (N × 6.25); they were also analyzed for crude fat (EE—ether extract) content (SOXTHERM, C. Gerhardt GmbH, Königswinter, Germany) [20]. Cell wall fractions NDF (neutral detergent fiber) and ADF (acid detergent fiber) were also determined via the method described by Van Soest et al. [21] using the ANKOM 220 analyzer (ANKOM Technology Corporation, Fairport, NY, USA).

Fat, protein, lactose, and solid non-fat (SNF) of milk were determined using a MilkcoScan (TUV.CERT/Milcotronic, Sofia, Bulgaria) standardized for goat milk, with automatic infrared analysis. The MUL (milk urea level) was determined using an enzymatic method [22].

#### 2.3.2. Milk Cholesterol Analysis

The method described by Cozma et al. [12] was used for the analysis of milk cholesterol. Briefly, the fat was extracted with a mixture of chloroform and methanol (2:1). After the mixture was stirred and washed in a separatory funnel, the organic phase was dried over anhydrous Na_2_SO_4_, evaporated to dryness, and taken up in chloroform. Cholesterol concentration was determined in two steps: cholesterol derivatization followed by gas-chromatographic separation. BSTFA (bistrimethylsilyl-trifluoroacetamide) and TMCS (trimethylchlorosilane) (2:1) were used for cholesterol derivatization, and the mixture was kept for 2 h at 60 °C. The obtained solution and standards were injected into a Varian 3800 GC-4000 gas chromatograph (Varian, Inc., Palo Alto, CA, USA) equipped with a flame ionization detector (FID) and a CP-Sil 5CB column (Varian Inc., Palo Alto, CA, USA) (25 m × 0.25 mm × 0.25 μm). Helium was used as the carrier gas, applying a flow rate of 1.9 mL/s. The operating parameters of the gas chromatograph were as follows: the injector and detector temperatures were 260 and 290 °C, respectively; the oven temperature was 130 °C at the beginning (maintained 3 min), then increased 10 °C/min to 200 °C (maintained 3 min), and further increased 20 °C/min to 290 °C (maintained 5 min). Quantitative determination of cholesterol in milk samples was carried out using the internal standard (Sigma-Aldrich) by comparing the areas obtained for milk samples and, respectively, for cholesterol of known concentration.

#### 2.3.3. Feed and Milk Fatty Acid Analysis

To obtain and quantify FA methyl esters (FAMEs) from pasture (shrubs and grass), hay (grass hay and alfalfa hay), hemp seeds, and concentrates (without H and with Hs), we used the method described by Mierlita et al. [23].

The milk fat was extracted according to the method described by Folch et al. [24] and transmethylation was performed with methyl alcohol. The lipid extract was treated with benzene, BF3, and methanol, after which it was heated and maintained at a temperature of 80 °C for 2 h. Next, hexane and distilled water were added and mixed with vortexing, and the organic phase obtained was treated with anhydrous Na_2_SO_4_. After treating the samples once more with hexane and distilled water, they were vortexed, and the extract was brought to dryness in a rotary evaporator. Finally, the obtained residue was treated with hexane.

The separation and quantification of FAME from feed and milk samples was performed using a Varian 3800 GC-4000 gas chromatograph (Varian, Inc., Palo Alto, CA, USA) equipped with a flame ionization detector (FID) and a column CP-Sil 88 (100 m × 0.25 mm × 0.20 µm; Varian, Inc., Palo Alto, CA, USA). Helium was used as a carrier gas, applying a flow rate of 1.9 mL/s. The injector and detector temperatures were 250 and 260 °C, respectively. The initial oven temperature was programmed at 40 °C (held for 3 min), then increased by 15 °C/min to 200 °C and held in the tray for 3 min at 200 °C. The temperature was then increased again by 20 °C/min to 240 °C and held steady for 5 min.

FAMEs were identified by comparing their retention times with those of pure methyl ester standard (Supelco 37 Component FAME Mix, Sigma-Aldrich, St. Louis, MO, USA). Individual FA concentrations were expressed as a % of total identified FAs.

#### 2.3.4. Milk Fat-Soluble Vitamins Analysis

Fat-soluble vitamins were extracted from milk according to the method described by Santa et al. [19]. The determination of retinol and α-tocopherol content in milk was performed with an HPLC (Agilent Technology series 1100, Santa Clara, CA, USA) equipped with a Phenomenex SphereClone column (5 µm, 150 × 4.6 mm). The mobile phase was methanol at a flow rate of 1.3 mL/min, and the detector was set to 292 nm for α-tocopherol and 340 nm for retinol. High purity standards (R7632 and T3251; Sigma Aldrich, Madrid, Spain) were used for quantification. Duplicates were analyzed for each sample.

#### 2.3.5. Milk Antioxidant Capacity Analysis

The TAC of whole-milk samples was determined using the ABTS method described by Mierlita et al. [23]. A 2,2′-azinobis (3-ethylbenzthiazoline-6-acid) (ABTS) solution was dissolved with potassium persulfate (2.45 mM), and the mixture was kept in the dark for 12–14 h. By diluting the ABTS^•+^ solution with methanol, an absorbance of 0.70 ± 0.02 at 730 nm was obtained. The milk sample (0.01 mL) was treated with an ABTS^•+^ solution (1 mL), and the removal of the ABTS radical was monitored using spectrophotometry. As a standard, we used the water-soluble vitamin E analog Trolox, and the TAC value was calculated based on the calibration curve. All analyses were performed in duplicate.

### 2.4. Calculations and Nutritional Indices

The fat and protein-corrected milk (FPCM) and energy-corrected milk (ECM) were calculated from the daily milk yield and milk composition, using the equations [25,26]:

FPCM (kg/d) = Milk yield (kg/d) × (0.26 + 0.1352 × Fat (%) + 0.079 × CP (%))(1)

ECM (kg/d) = Milk production (kg/d) × (0.38 × Fat (%) + 0.24 × CP (%) + 0.17 × lactose (%))/3.14 (2)

Based on the fatty acid composition of milk fat, the nutritional indices and FA ratios related to healthy fat consumption were calculated [1,13]:

AI (Atherogenicity Index) = [C12:0 + (4 × C14:0) + C16:0]/UFA(3)

TI (Thrombogenicity Index) = (C14:0 + C16:0 + C18:0)/[(0.5 × MUFA) + (0.5 × n-6 FA) + (3 × n-3 FA) + (n-3/n-6 FA)](4)

h/H (hypocholesterolemic:Hypercholesterolemic FA ratio) = (C18:1 c-9 + PUFA)/(C12:0 + C14:0 + C16:0)(5)

HPI (Health-promoting Index) = UFA/(C12:0 + (4 × C14:0) + C16:0)(6)

NVI (Nutritive Value Indices) = (C18:0 + C18:1)/C16:0(7)

PI (Polyunsaturation Index) = C18:2 n-6 + (C18:3 n-3 × 2)(8)

DFA (Desirable FA) = C18:0 + UFA(9)

### 2.5. Statistical Analysis

A 2 × 2 factorial experimental design was used, with two types of forage (hay vs. mixed shrubs–grass rangeland) and two types of concentrates (standard concentrate without Hs vs. experimental concentrate containing Hs). The two-way analysis of variance (ANOVA) using a general linear model (GLM) of the SAS [27] was carried out to determine the effect of diets on milk production, fatty acid composition, antioxidant content, and total antioxidant capacity of milk. The fixed effects were forage type, hemp seed supplementation, and interactions between fixed effects. The linear model used was as follows:

Yijk = µ + Fi + Hsj + (F × Hs)ij + εijk

where Yijk refers to observations for dependent variables; µ is the overall mean; Fi the fixed effect of forage type (i), hay or mixed shrubs–grass rangeland); Hsj is the fixed effect of hemp seeds (j), without or with hemp seeds); F × Hs is interactions between among these factors; and εijk is the random effect of the residual. Significance between individual mean was identified using Tukey’s multiple-range tests. Means were declared statistically different when *p* < 0.05. All data were presented as means with a pooled standard error of the mean estimates. To determine the Pearson correlation coefficients (r) between the different milk variables (TAC, major FA content, fat-soluble vitamin content), we used the CORR procedure of SAS.

## 3. Results

### 3.1. Forage and Concentrate Nutritive Characteristics

The chemical composition and the content of major FAs in the main species of shrubs and grasses in the pasture, as well as of the other fodder used in the diet of the goats (pasture hay, alfalfa hay, hemp seeds, and concentrates), are presented in Table 1 and Table 2, respectively.

Hemp seed (Hs) had the highest CP content (254.8 g/kg DM), while hay (pasture hay and alfalfa hay) had the highest content of NDF and ADF. Among the shrub species, acacia (*Rubinia pseudacacia*), which was predominant in the pasture, had the highest CP content, which was even higher than that of alfalfa hay (228.2 g/kg DM vs. 169.7 g/kg DM), while the content of NDF and ADF was similar in the two feeds (Table 1 and Table 2).

Pasture grass, shrubs, and hay were good sources of ALA, while the concentrate was a rich source of LA. Hemp seeds had the highest crude fat content (33.11% of DM), and were an important source of LA (54.80% of total FA) but also of ALA (18.63% of total FA). The introduction of Hs in the concentrate mixture led to an increase in total PUFA content, but especially ALA, by 2.5 times compared to the standard concentrate (9.47% vs. 3.88% of total FA) (Table 2).

Table 2 reports the composition of the two types of concentrates used in goat feed. The standard concentrate (concentrate C) was characterized by a higher inclusion of maize grain (55.0 vs. 47.0% as fed, for concentrate C vs. concentrate Hs), rapeseed meal (21.0 vs. 11.0% as fed, for C vs. Hs), and sunflower meal (12.0 vs. 5.0% as fed, for concentrate C vs. concentrate Hs), which were partially replaced by hemp seeds in the experimental concentrate (concentrate Hs).

### 3.2. Daily Milk Yield and Composition

The milk production and chemical composition are shown in Table 3. Daily milk production during the experimental period was higher (*p* < 0.01), and milk fat content (*p* < 0.001) and total solids (*p* < 0.05) were lower. Goats that grazed on mixed shrubs–grass (SG goats) gave on average lower yield of milk (milk ECM: energy-corrected milk; FPCM: fat and protein-corrected milk), protein (*p* < 0.05), and lactose (*p* < 0.05) than hay-fed goats (H goats) (Table 3). The urea content of milk was higher (*p* < 0.01) in SG than in H. Supplementing the diet with Hs (groups H-Hs and SG-Hs) caused a significant increase in daily milk production (*p* ˂ 0.05), milk fat content (*p* < 0.001), and total solids (*p* < 0.05).

The dietary treatments tested in this study did not affect the content of protein, lactose, or SNF (total solids non-fat) in milk (*p* ˃ 0.05) (Table 3).

The milk cholesterol concentration was lower in goats that had access to mixed shrubs–grass rangeland compared to those fed indoors with hay-based diets (*p* ˂ 0.01). The introduction of Hs into the diet of goats had an effect of lowering the cholesterol concentration in milk (*p* ˂ 0.05).

### 3.3. Milk Fatty Acid Profile and Health Indices

Table 4 and Table 5 show the FA content of milk fat according to dietary treatment. With few exceptions, almost all milk FAs were significantly affected by the dietary treatments. The main FAs in goat milk were C14:0, C16:0, C18:0, and C18:1c-9, each accounting for more than 8% of total FAs. The type of basic feed did not influence the content of milk in C14:0 and C18:1 c-9 (*p* ˃ 0.05); instead, the concentration of C16:0 was higher in goats fed indoors with hay (groups H), and the concentration of C18:0 was higher in goats fed on mixed shrubs–grass. Feed supplementation with Hs decreased the content of C14:0 and C16:0 and increased C18:0 and C18:1 c-9 in milk (*p* ˂ 0.01) (Table 4).

Goats that grazed on mixed shrubs–grass produced, on average, milk with a lower concentration of medium-chain FAs (C10:0, C16:0, *p* < 0.05) and a higher concentration of C18:0 (*p* < 0.001), C18:1 t-11 (*p* ˂ 0.001), C18:2 n-6 (*p* < 0.05), CLA c-9,t-11 (*p* ˂ 0.01), C18:3 n-3 (*p* ˂ 0.01), C20:5 n-3 (*p* ˂ 0.01), and C22:5 n-3 (*p* ˂ 0.05) than goats fed hay. The introduction of Hs into the diet of goats resulted in a lower proportion of medium-chain FAs in milk fat (C10:0, C12:0, C14:0, C16:0) and a higher proportion of C18:0 (*p* < 0.01), C18:1 t-11 (*p* < 0.001), C18:1 c-9 (*p* ˂ 0.001), C18:2 n-6 (*p* ˂ 0.05), CLA c-9,t-11 (*p* ˂ 0.001), C18:3 n-3 (*p* ˂ 0.001), C20:5 n-3 (*p* ˂ 0.01), and C22:5 n-3 (*p* ˂ 0.01) compared to goats fed the standard concentrate mixture (Table 4).

Grazing, compared to indoor feeding but also supplementing the diet with Hs, had the effect of decreasing the proportion of SFAs and increasing PUFA concentrations in goat milk fat (*p* < 0.01). Thus, the milk with the lowest proportion of SFAs (62.49% of total FAs) and the highest concentration of PUFAs (9.67% of total FAs) was obtained from goats that grazed on pastures dominated by shrubs and bushes and whose diet was supplemented with Hs (SG-Hs group) (Table 5). The proportion of FAs with a hypercholesterolemic effect (HFA = 12:0 + 14:0 + 16:0) in milk fat was lower (*p* ˂ 0.05), and the proportion of FA with a hypocholesterolemic effect (hFA = C18:1 *cis* + PUFA) was higher (*p* ˂ 0.01) in SG goats than in H goats and in goats supplemented with Hs than in those that received the standard concentrate (Table 5). Thus, the lowest concentration of HFA (33.64% of total FA) and the highest concentration of hFA (30.16% of total FA) was found in goats that grazed and received a daily supplement of 200 g Hs (SG-Hs group), respectively.

**Figure 1:**
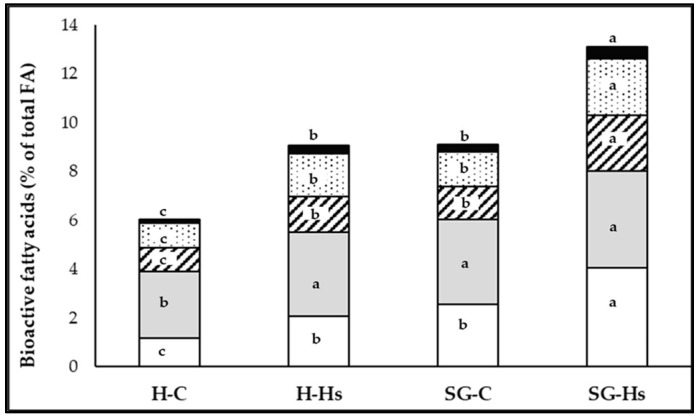
The effect of the diet based on hay (H) or shrub–grass mixture (SG) with hemp seeds (H-Hs and SG-Hs) and without (H-C and SG-C) on the bioactive FA content of milk fat. Inside each bar: C18:1 *trans*-11 (white area); C18:2 *cis*-9, *cis*-12 (gray area); CLA *cis*-9, *trans*-11 (line area); C18:3 *cis*-9, *cis*-12, *cis*-15 (dotted area); C20:5 n-3 + C22:5 n-3 (black area); a,b,c = between bars the letter within the same colour differ at *p* < 0.05.

Dietary supplementation with Hs induced an overall increase in C18:1 *trans* and CLA c-9,t-11; however, the response to Hs supplementation varied according to the type of forage in the diet, with greater increases in grazing goats than in hay-fed goats (*p* ˂ 0.05). Higher proportions of healthy FAs (VA, CLA, ALA, EPA, and DPA) were found in the milk fat of pastured goats than those fed indoors with hay, but also in goats that received Hs in feed compared to those that received the standard concentrate (Figure 1). Thus, the highest concentrations of healthy FAs in milk fat were obtained in SG-Hs goats (% of total FA): VA—4.05%; CLA c-9,t-11—2.29%; ALA—2.32%; EPA—0.20%; and DPA—0.28% (Table 4).

Significant interactions between the type of dietary staple and dietary Hs supplementation were identified for C18:0, C18:1 t-11, C18:1 c-9, CLA c-9,t-11, and C18:3 n-3 content (*p* < 005) (Table 4) but also for *cis*-total FA and hypocholesterolemic FA (*p* < 0.05) (Table 5).

**Figure 2:**
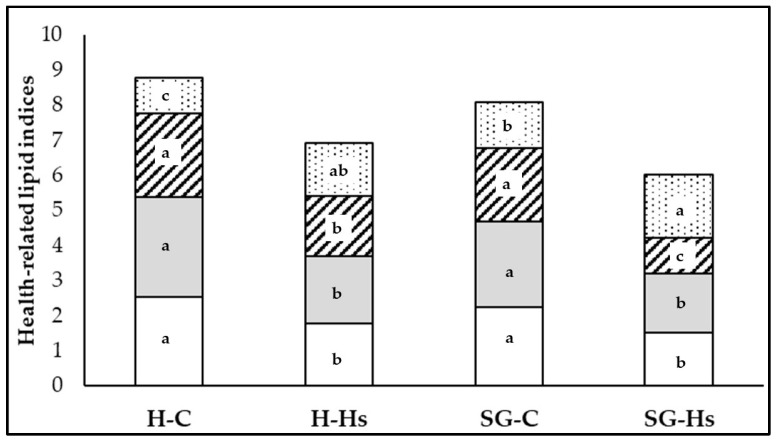
The effect of the diet based on hay (H) or shrubs–grass mixture (SG) with hemp seeds (H-Hs and SG-Hs) and without (H-C and SG-C) on the health-related lipid indices of milk. Inside each bar: n-6/n-3 FA (white area); AI (atherogenic index) (gray area); TI (thrombogenicity index) (lines area); NVI (nutritive value indices) (dotted area); a,b,c = between bars the letter within the same colour differ at *p* < 0.05.

The n-6/n-3 FA ratio and atherogenicity indices (AI) and thrombogenicity indices (TI) were significantly lower in the milk of SG goats compared to milk obtained from H goats, but also in goats that received Hs compared to those that received the standard concentrate (Figure 2).

The best values for the ratio of n-6/n-3 FA, AI, and TI, from the point of view of human health, were found in the milk of SG-Hs goats, and the worst values were found in conventionally produced milk (H-C: goats fed hay and standard concentrate). In addition, when goat grazed on mixed shrubs–grass rangeland and supplemented the diet with Hs, respectively, the h/H FA ratio (C18:1 *cis* + PUFA/12:0 + 14:0 + 16:0) in milk was significantly improved (Table 6).

Higher values of HPI (health-promoting index), NVI (nutritive value indices), PI (polyunsaturation index), and DFA (desirable FA) were found in SG milk compared to H, and the supplementation of diets with Hs significantly (*p* ˂ 0.01) increased their value, reflecting an improvement in the quality of milk fat in terms of the effect on human health.

### 3.4. Vitamins Content and Antioxidant Activity of Milk

**Figure 3:**
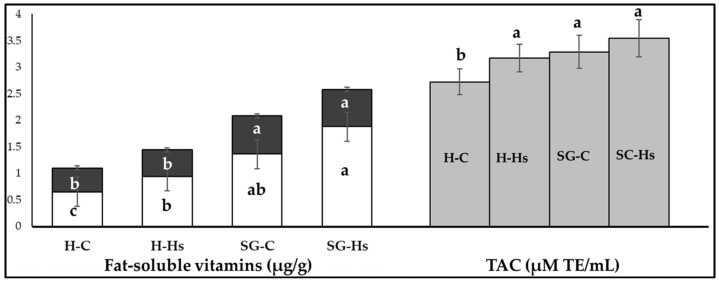
The effect of the diet based on hay (H) or shrubs–grass mixture (SG) with hemp seeds (H-Hs and SG-Hs) and without (H-C and SG-C) on the soluble vitamins content (α-tocopherol: with white color on bar; retinol: black area on bar) and antioxidant capacity (TAC) of milk stored for 4 days at 2 °C. a,b,c = between bars the letter within the same colour differ at *p* < 0.05.

The content of milk in α-tocopherol and retinol was significantly higher in the SG group compared to the H group (*p* ˂ 0.01), both for raw and pasteurized milk (Table 7) and for stored milk (Figure 3). Supplementing the diet with Hs increased α-tocopherol concentrations and TAC value in milk. The concentrations of these vitamins (α-tocopherol and retinol) in milk were negatively affected by the time the milk was stored in the refrigerator.

The value of the total antioxidant capacity (TAC) of milk was higher for milk obtained from grazing goats than for those fed indoors with hay (SG ˃ H), as well as for those supplemented with Hs, so the highest TAC value was obtained in SG-Hs goats (4.28 µM Trolox equivalents/mL milk) (Table 7; Figure 3). The pasteurization of milk did not change the TAC value of milk, while storing milk for 4 days in the refrigerator reduced the antioxidant activity of milk.

Pearson’s correlation showed that C18:3 n-3 was positively correlated with the total C18:1 (r = 0.759, *p* < 0.001), C18:1 t-11 (r = 0.883, *p* < 0.001), CLA c-9,t-11 (r = 0.906, *p* < 0.001), retinol (r = 0.474, *p* < 0.01), and α-tocopherol (r = 0.815, *p* < 0.001). On the other hand, TAC was negatively correlated with the concentration of C18:3 n-3 (r = 0.528; *p* < 0.001) and C18:2 n-6 (r = 0.380, *p* < 0.01) and positively correlated with the retinol content of milk (r = 0.358, *p* < 0.01) and α-tocopherol (r = 0.615, *p* < 0.001) for all types of milk (Table 8).

**Figure 4:**
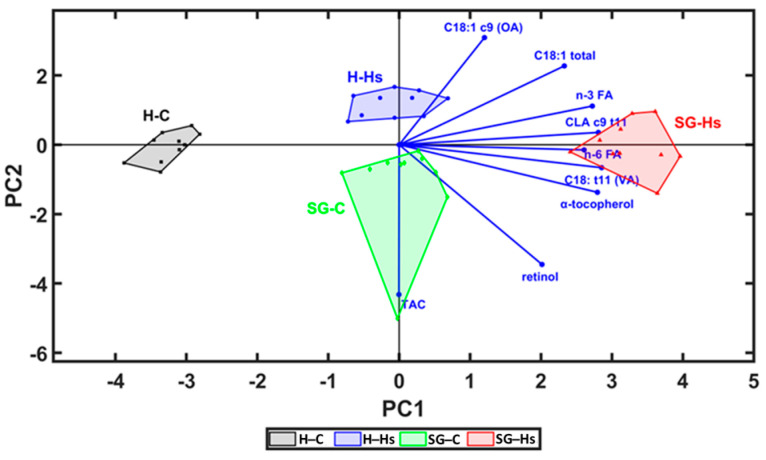
Principal component analysis (PCA) loading plot which presents the relationship between milk content in bioactive fatty acids, antioxidants, and antioxidant capacity of milk.

**Figure 5:**
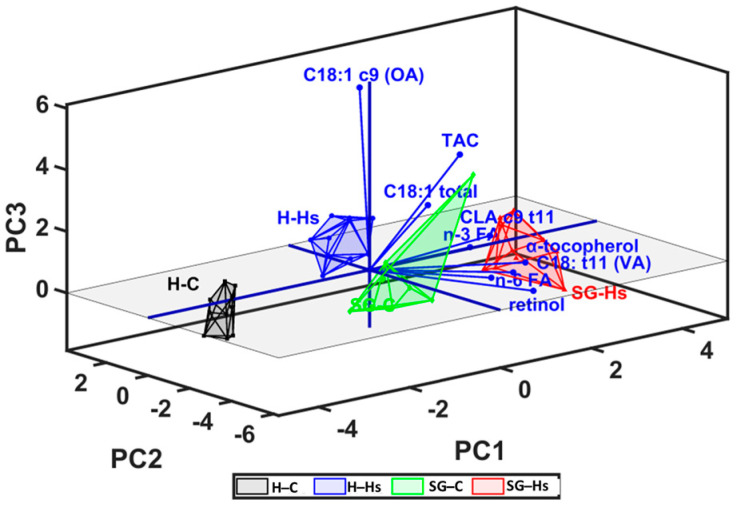
Score and loading plot of principal component analysis (PCA) for the PC1 (first principal component) vs. PC2 (second principal component) vs. PC3 (third principal component) of bioactive compounds and antioxidant capacity of milk, which show the separation of SG-Hs milk to H-C, SG-C, and H-Hs milk.

The milk content in bioactive FA (VA, CLA c-9 t-11, n-3 FA, and n-6 FA), fat-soluble vitamins and TAC were analyzed by PCA (principal component analysis) in Unscrambler. This combination of PCs was selected because it allowed the best separation of groups (Figure 4 and Figure 5). In this loading plot, it can be observed that SG-Hs milk had the highest concentration of n-3 FA, CLA c9,t11, VA, and fat-soluble vitamins and a higher antioxidant activity, which is confirmed in Table 4, Table 5 and Table 7. In addition, the loadings for PC2 divide the H-Hs and SG-C milk, showing positive loadings for supplementing the diet with Hs and negative loadings for the use of mixed shrubs–grass rangeland with standard concentrate. In the loading of the correlation graph (Figure 5), it can be see that the milk content in VA, CLA c9,t11, n-3 FA, n-6 FA, and tocopherol could be identified as factors which contributed more significantly to the variation between milk samples, via the PC2 vector. The distribution and correlation between the milk content in bioactive FA, antioxidants, and the TAC of milk were confirmed by statistical analysis (Table 8).

## 4. Discussion

### 4.1. Milk Yield and Components

In the present research, milk production was lower than that of other breeds of dairy goats but close to that reported in the literature for goats from the local Carpathian breed [7,12,28]. The milk fat, protein, and lactose contents were similar to those reported by Anghel et al. [28] for goats of the same breed but with very different diets. Thus, neither mixed shrubs–grass rangeland nor Hs dietary supplementation negatively affect milk production and composition in Carpathian goats.

Even though the nutritional quality of the pasture (mixed shrubs–grass rangeland) was slightly better than that of hay (Table 1 and Table 2), milk production was lower in SG than in H (Table 3). Thus, the most important factor driving differences in milk production between SG and H would be energy expenditure for locomotion on pasture. Based on walking distances, Steinshamn et al. [18] calculated that goats grazing on woodland rangeland expended an average of 5.8 to 9.0 MJ NEL daily for locomotion. In addition, woody pastures often contain high amounts of tannins, which can reduce feed digestibility and utilization [18].

The higher fat content of SG milk than H may be due to lower milk production in SG goats, which lead to an increase in the level of components in milk [7]. On the other hand, it may be due to the mobilization of body fat in SG goats due to a lower energy balance in the diet. In this regard, relevant studies have shown that there is a correlation between the lower energy balance of the diet and the high fat content of goat milk [18,29].

The study previously carried out by Min et al. [30] also found a similar milk protein content but lower milk urea content in grazing goats than hay-fed goats. In the present study, the higher milk urea content in SG than in H indicates that the balance between energy and protein for milk protein synthesis was probably more optimal in goats fed with hay than those fed on pasture. Contrary to the results obtained in this study, Bodnár et al. [31] concluded that grazing goats on a native-grass-only pasture had a beneficial effect on milk protein content and milk-to-cheese efficiency compared to goats fed indoors with canned forage.

In the present study, milk production and milk fat and total solids content increased when goats were fed Hs-supplemented diets (Table 3), in agreement with the results previously obtained by Mierlita [11,13] in dairy sheep. Cremonesi et al. [32] reported that the introduction of hemp seeds into the diets of goats (9.4% of DM) did not change milk production; instead, the milk fat content increased significantly (from 2.84 to 3.55%). Similarly, Emami et al. [33] found that supplementing goat diets with 2.5% pomegranate or linseed oil increased milk fat content.

The FA profile of milk fat is majorly influenced by the degradability rate of dietary fat in the rumen. In this regard, oilseeds with a high ruminal degradability (e.g., rapeseed) negatively affect milk fat content, unlike oilseeds that have a low ruminal degradability (e.g., flaxseed) [34]. Therefore, it can be assumed that hemp seeds have a low ruminal degradability and slowly release unsaturated FAs in the rumen, reducing the amount of FAs transformed in the rumen via biohydrogenation, favoring the increase in milk fat [35].

Dietary supplementation with Hs did not affect milk protein content (Table 3), which is in agreement with other reports [36,37] but different from the results reported by Cozma et al. [12], where supplementing the diet with hemp oil had a positive effect.

In the present study, dietary supplementation with Hs led to a significant decrease in milk cholesterol levels (Table 3), contrary to Cozma et al. [12], who reported that dietary supplementation with hemp oil had no influence on the concentration of cholesterol in goat milk. Results similar to those obtained in this study were reported in Holstein cows, when dietary supplementation with vegetable oil (soybean oil, rapeseed oil) decreased the milk cholesterol concentration [38].

### 4.2. Fatty Acid Composition of Milk

Although many studies in the literature have shown that grazing increases milk MUFAs and PUFAs and decreases SFAs compared to hay feeding [11,13,18,19,39,40], in the present study, the proportion of MUFA in milk was not affected by the type of basic forage in the goats’ diets (Table 4 and Table 5). The difference could be explained by the good quality of the hay diet, which included concentrates and induced a healthier fatty acid profile in the milk fat [41]. Similar to the results obtained in this study, De Lucena et al. [42] found a higher proportion of UFA and lower SFA in the milk of goats fed tannin-rich diets, because tannins from woody pastures reduced FA biohydrogenation in the rumen. In addition, it is known that increasing the flow of unsaturated FAs into the mammary gland reduces the proportion of short- and medium-chain saturated FAs in milk fat by reducing acetyl-CoA carboxylase activity [43]. The presence of tannins in woody pastures probably affected nutrient absorption and minimized de novo FA synthesis in the mammary gland, accentuating the mobilization of FA from adipose tissue in the goats’ bodies [10]. Thus, the milk of SG goats had lower concentrations of short- and medium-chain FAs (mainly C16:0) and increased proportions of long-chain FAs, mainly C18:0 and C18:1, which originate from adipose tissue mobilized from the body [10].

Grazing is known to increase the concentration of FAs that have positive effects on human health (in particular, C18:1 t-11, CLA c-9,t-11, and n-3 FA) in milk fat [11,13,18,19]. In the present study, the higher proportion of healthy FAs (C18:1 t-11, CLA c-9,t-11, C18:3 n-3, C20:5 n-3, and C22:5 n-3) in milk produced in SG than in H can be explained by a higher supply of precursors (C18:2 n-6 and C18:3 n-3) present in the pasture (shrubs–grass mixed pasture) than in hay. In addition, the high content of tannins in woody pastures affects the biohydrogenation processes of dietary FA in the rumen. For example, the high content of tannins from woody pastures caused an increase in the proportion of LA and ALA in sheep milk [44]. The alteration of the ruminal biohydrogenation process due to tannins from woody pastures could also explain the higher concentration of long-chain n-3 FA (EPA and DPA) resulting from the conversion of dietary ALA (C18:3 n-3) [45].

Although grazing increases the intake of UFA and mainly PUFA [39,40], in our study, SG goats had a higher content of C18:0 and similar MUFA content in milk fat with H goats, particularly as a result of higher C18:1 in the milk of SG goats (Table 4 and Table 5). Previous studies reported a higher rate of FA biohydrogenation (C18:1 → C18:0) for fresh feed [46], which negates the effect of higher PUFA consumption in SG goats. A higher amount of substrate (C18:0) determined a higher proportion of C18:1 in the milk of SG goats due to the fact that a part of C18:0 through ∆9-desaturase activity is converted to C18:1 [39]. The higher proportion of C18:0 in SG milk than in H milk could also be explained by the lower desaturation of C18:0 into C18:1 in the mammary gland, as shown by ∆9C18 activity (Table 5).

The content of RA (rumenic acid) was higher in the milk of pasture-fed goats, which agreed with the results reported by Decandia et al. [47], who found a higher RA in the milk of goats grazing on a shrub-dominated pasture than in the milk of goats fed indoors with canned forage. Grazing has been shown to significantly improve the development of rumen bacteria (such as *Butyrivibrio fibrisolvens*), with a positive effect on the production of VA and CLA isomers [39]. VA is converted to RA (CLA c-9,t-11) by Δ9-desaturase in the mammary gland and some human tissues [48].

The milk of goats supplemented with Hs (H-Hs and SG-Hs) had lower proportions of SFA (mainly FAs with atherogenic effects: 12:0, 14:0, and 16:0) and higher concentrations of MUFAs (mainly 18:1 c-9 and 18:1 t-11) and PUFAs (mainly RA and ALA) than the milk of the groups that received a standard concentrate (Table 4 and Table 5), as reported by previous studies, when the goats’ diet was supplemented with hemp oil [11] or linseed [37,49].

Supplementation of the diet with Hs decreased the proportion of de novo synthesized FA (C10:0, C12:0, C14:0, and C16:0) in milk fat, as the increased amount of long-chain FAs reduced the activity of acetyl -CoA carboxylase in the mammary gland [50].

The high proportion of MUFA found in the milk fat of goats supplemented with Hs can be related to the higher intake of LA from Hs, which, through rumen biohydrogenation, led to a significant increase in the proportion of oleic acid (C18:1 c-9, *p* < 0.001). On the other hand, the high biohydrogenation of dietary C18:2 n-6 in the rumen in goats supplemented with Hs is also supported by the higher proportion of C18:0 compared to milk from goats that did not receive Hs feed (H-C and SG-C groups) (*p* < 0.001).

The higher level of PUFA in the milk of goats supplemented with Hs was due to the higher levels of LA and ALA in Hs (Table 2). The high proportion of ALA in Hs ensured higher concentrations of n-3 FAs (ALA, EPA, and DHA) in milk fat. The concentration of ALA found in goat milk in our research was considerably higher (1.42% of total FA) than the value reported by Zan et al. [51] (0.88%) in goats grazing on alpine pasture, but the total number of FAs identified in their study was higher. However, Cozma et al. [12] reported that dietary supplementation of goats with 4.7% hemp oil did not affect the concentration of ALA in milk fat. The lack of an increase in the concentration of ALA in milk fat compared to the present study could be attributed to the fact that ALA was provided in the form of oil (a free form) that was vulnerable to rumen biohydrogenation.

Hemp seeds, due to their high content of ALA (18.63% of total FA) compared to other vegetable oils (<9%, except for linseed [6]), ensured a high content of n-3 FAs (ALA, EPA, and DPA) in goat’s milk, which have beneficial effects on human health. ALA in ruminant feed, mainly in the mammary gland, undergoes a series of elongation and desaturation reactions, leading to the formation of long-chain PUFAs (EPA, DPA, and DHA) [2]. The high LA and ALA content of Hs (Table 2) probably contributed to the higher proportion of VA and CLA c-9,t-11 (RA, rumenic acid) in milk fat. In fact, RA comes from the ruminal biohydrogenation of dietary LA and ALA but also from the endogenous synthesis carried out in the mammary gland via ∆9-desaturase, starting from VA. However, ∆9-desaturase activity (calculated as product/product + substrate) did not change significantly in the Hs-supplemented groups (Table 5), probably due to the increased availability of PUFA in the mammary gland [29]. Thus, we can speculate that the higher concentration of CLA c-9,t-11 in the milk of goats supplemented with Hs is due to the higher intake of the two precursors (C18:2 n-6 and C18:3 n-3) that were biohydrogenated in the rumen. In addition, fats containing large amounts of C18:2 n-6 and C18:3 n-3, as is also the case with Hs, have an inhibitory effect on cellulolytic bacteria in the rumen [52], reducing the process of biohydrogenation of the two unsaturated FAs, and thus could positively influence the levels of rumen biohydrogenation intermediates in milk.

The ∆9-desaturase activity (∆9C14 and ∆9C16) decreased significantly when goats were fed Hs-supplemented diets, while the desaturase index (DI) increased compared to unsupplemented diets (Table 5). A reduction in Δ9-desaturase and an increase in DI were observed in goats supplemented with linseed or soybean oil [53] and in sheep supplemented with hemp seed or hempseed cake [11].

In this study, the positive correlation of ALA with total C18:1, C18:1 t-11, and CLA c-9,t-11 confirms its biohydrogenation to C18:1 *trans* isomers. In addition, the correlation of ALA with C18:1 c-9 and CLA c-9,t-11 suggests their formation via the desaturation of C18:0 in the mammary gland.

### 4.3. Health-Related Lipid Indices

The sanogenic lipid indices of milk fats found in the current study fell within the values presented in the specialized literature for goat milk [45]. The quality of milk fat is frequently evaluated based on the PUFA/SFA ratio, which should have a value above 0.45 for the prevention of cardiovascular disease and other chronic diseases [54]. In the present study, using mixed shrubs–grass rangeland but also supplementing the diet with Hs led to a significant increase (*p* < 0.001) in the PUFA/SFA ratio in milk. However, the values of the PUFA/SFA ratio were much lower than those recommended for the human diet (0.07–0.15) (Table 6).

Nutritionists recommend foods with a low n-6/n-3 FA ratio to reduce the risk of hypertension, cardiovascular disease, diabetes, and cancer in humans [55]. In the current study, the n-6/n-3 FA ratio of goat milk was within the recommended values (<4) for human health in all treatment groups, but the best values were obtained in goats supplemented with Hs.

The atherogenic index (AI) describes the relationship between SFAs, which are considered pro-atherogenic, and UFAs, which are considered anti-atherogenic. The thrombogenicity index (TI) assesses the propensity of FA to form clots in blood vessels. AI and TI values lower than three are considered beneficial for human health [55]. In the present study, all milk samples fell within the recommended value; the lowest values for AI and TI were found in SG-Hs milk (goats that grazed and whose diets were supplemented with Hs), and the highest values were found in conventionally produced milk (H-C: goats were fed hay and standard concentrates). In agreement with the current study, De Lucena et al. [52] reported a decrease in AI and TI in goat milk when the diet was high in tannins (28 g tannins per kg DM); woody pastures are known for their high tannin content. The health-promoting index (HPI) is considered the inverse of TI, so milk with a high HPI value is healthier for humans [54]. In the present study, higher HPI values were found in SG milk, and these were within the range reported by Claps et al. [56] (0.34–0.45) for pasture-raised goat milk. The supplementation of diets with Hs significantly increased (*p* < 0.01) the HPI value (0.49–0.54).

The h/H FA ratio describes the relationship between FAs with a hypocholesterolemic effect and those with a hypercholesterolemic effect, so high values for this ratio are desirable. A higher h/H ratio was found in the milk of goats supplemented with Hs. In our study, the h/H FA ratio was in accordance with the results previously published by Chen and Liu [54] and Bodnár et al. [31], who found higher values for milk obtained on pastures consisting of different herbaceous species compared to milk obtained indoors with diets based on canned forages. The desirable FA (DFA) represents the sum of anti-atherogenic fatty acids (UFAs + C18:0) that lower plasma cholesterol and triacylglycerols [54]. Thus, higher values are preferred for DFA. Higher values for DFA were found in the milk produced by SG goats (*p* < 0.01) but also when the goats’ diet was supplemented with Hs (*p* < 0.001) (Table 6).

### 4.4. Fat-Soluble Vitamins and Antioxidant Capacity

Natural antioxidants in milk are important for human health because they can neutralize and eliminate free radicals and their harmful effects [57]. Uncontrolled free radicals can lead to atherosclerosis, cardiovascular disease, diabetes, cancer, and the breakdown of some biochemical compounds in the diet. The results of this study showed that SG milk has a higher content (*p* < 0.01) of natural antioxidants (α-tocopherol and retinol) than H milk (Table 7). These results agree with those previously reported by Delgado-Pertínez et al. [46] regarding the α-tocopherol content of milk obtained from goats grazing on Mediterranean shrublands, although in our study, the values were higher, probably due to the different botanical composition of the mixed shrubs–grass rangeland, the vegetation stage of the plants, and probably differences in the leaf:stem ratio or environmental factors. The lower content of α-tocopherol and retinol in H milk is due to the wilting and drying of plants, which causes oxidative degradation of these nutrients [58].

The supplementation of the diet with Hs did not change the retinol content of milk, although Hs are a β-carotene form [11]. The results obtained in this study are in agreement with Cozma et al. [12], who supplemented goats’ diets with hemp oil, but in disagreement with Puppel et al. [59], who showed that supplementing the diet with flaxseed increased milk retinol concentration in cows. The differences between these studies could be due to a species effect (goat vs. cow) or the nature of the fat supplement (hemp vs. flax). Unlike cows, goats convert all the β-carotene in their feed into retinol, so their milk has more vitamin A, which is comparable from this point of view to the milk of humans [60].

The results of this study demonstrate that the total antioxidant capacity (TAC) of goat milk is positively correlated with the content of α-tocopherol and retinol, since these vitamins intervene in the capture of free radicals and the inhibition of lipid peroxidation [61]. In addition, high-fat milk has been shown to have a higher antioxidant capacity, as measured using the ABTS assay, than low-fat milk due to its higher content of lipophilic vitamins; positive correlations have been established between the fat content and the TAC value of milk [54].

Significant decreases in α-tocopherol and retinol content were observed during milk storage in all experimental variants, probably because these vitamins acted as a hydrogen donor, which led to the accumulation of lipid hydroperoxides in milk. This mechanism could be responsible for the significant decrease in the TAC of stored milk in all types of milk. The results of this study agree with those previously reported by Havemose et al. [62] for cow’s milk.

In contrast to the results of this study, Yilmaz-Ersan et al. [63] reported an increase in TAC in pasteurized milk (90 °C for 10 min), as measured using the ABTS test, compared to raw milk. Similarly, other studies have demonstrated that heating milk to over 100 °C increases the antioxidant capacity of milk because thiol groups formed by protein unfolding act as hydrogen donors [61]. In the present study, milk pasteurization was performed at a lower temperature, namely 63 °C, for 30 min, which may explain why pasteurization did not change the TAC value, in agreement with other recent studies [19,23,61].

After 4 days of refrigerated storage of milk, the TAC value decreased for all types of milk, suggesting that higher concentrations of α-tocopherol and retinol in milk do not prevent the oxidation of unsaturated FAs from milk fat but delay this process, increasing the oxidative stability of milk [16].

## 5. Conclusions

The results of this study clearly demonstrate that incorporation of hemp seeds in the diet of goats at a level of 200 g/day significantly improved the milk quality by increasing the concentrations of FA considered beneficial for human health (mainly n-3 FA, VA, and CLA c-9,t-11), increasing the natural antioxidants content (α-tocopherol and retinol) and the antioxidant capacity (TAC) of milk. Mixed shrubs–grass rangeland had similar effects in the improving of milk quality compared to hay-based diets. The feeding system of goats with mixed shrubs–grass rangeland and supplementing the diet with hemp seeds ensured the enrichment of milk with the best nutritional and functional quality, confirmed by the FA profile, antioxidant content, and the value of health-related lipid indices.

Further research is needed to determine whether hemp seeds have positive effects not only on milk production and quality but also on the metabolic profile and more importantly on animal health.

## Figures and Tables

**Table 1:** Chemical composition and major fatty acids (FA) of mixed shrubs–grass rangeland consumed by goats ^1^.

	Chemical Composition (% on DM Basis)					FA Major (% of Total FA)		
	DM	CP	EE	NDF	ADF	C16:0	C18:2 n-6 (LA)	C18:3 n-3 (ALA)
(a) Shrubs and bushes								
* Rubinia pseudoacacia*	416.51	22.82	4.72	49.53	37.66	22.54	17.31	46.29
* Carpenus betulus*	438.76	18.54	5.33	52.27	39.19	18.82	16.95	45.96
* Prunus spinosa*	438.62	12.31	5.09	41.25	38.22	18.34	16.59	42.65
* Fagus sylvatica*	415.58	15.78	6.83	50.32	40.40	24.85	20.72	41.87
* Rosa canina*	537.82	10.12	5.47	30.21	21.17	17.71	20.51	45.15
* Rubus fructicosus*	478.37	16.82	7.81	27.65	19.38	16.14	29.07	34.61
* Wild blackberry*	504.44	9.83	3.26	51.68	42.72	18.34	19.01	38.72
* Crataegus monogyna*	521.47	13.75	6.28	24.83	19.92	12.62	15.45	32.70
(b) Herbaceous species	358.72	16.18	3.42	49.86	35.64	23.91	24.68	40.57

^1^ n = 4; DM: dry matter; CP: crude protein; EE: ether extract; NDF: neutral detergent fiber; ADF: acid detergent fiber; c: *cis*; LA—linoleic acid; ALA—α-linolenic acid.

**Table 2:** Chemical composition and fatty acid profile of feeds administered to goats.

Parameters	Pasture Hay	Alfalfa Hay	Hemp Seed	Concentrates	
				C	Hs
Chemical composition (% on DM basis)					
DM (%)	88.47 ± 1.27	91.34 ± 1.73	91.16 ± 1.11	90.21 ± 1.37	91.07 ± 1.02
Crude protein (CP)	9.41 ± 0.54	16.97 ± 0.75	25.48 ± 1.12	16.66 ± 0.58	16.58 ± 0.71
Ether extract (EE)	1.83 ± 0.11	2.17 ± 0.17	33.11 ± 0.09	3.32 ± 0.14	10.97 ± 0.28
NDF	54.72 ± 2.07	48.75 ± 1.90	33.84 ± 1.54	25.62 ± 1.12	26.16 ± 1.17
ADF	32.63 ± 0.54	34.18 ± 0.61	21.76 ± 0.39	10.46 ± 0.35	12.03 ± 0.26
NE_L_ (MJ/kg DM) **	4.77	4.18	9.63	8.58	8.72
Fatty acids (% of total FA)					
Total FA (g/kg DM)	12.51 ± 1.32	15.26 ± 0.27	297.41 ± 11.4	35.55 ± 1.28	102.49 ± 2.58
C16:0	30.86 ± 0.49	31.78 ± 0.62	7.04 ± 0.88	22.51 ± 0.38	18.19 ± 0.61
C18:0	3.23 ± 0.17	10.07 ± 0.09	2.52 ± 0.07	2.73 ± 0.11	2.60 ± 0.18
C18:1 *cis*-9 (OA)	7.54 ± 0.41	3.95 ± 0.82	10.42 ± 0.29	24.77 ± 0.69	20.68 ± 0.77
C18:2 n-6 (LA)	17.50 ± 0.94	20.41 ± 0.47	54.80 ± 0.76	35.14 ± 0.54	39.25 ± 0.58
C18:3 n-3 (ALA)	25.86 ± 1.56	26.58 ± 1.71	18.63 ± 1.26	3.88 ± 0.95	9.47 ± 1.17
Saturated FA	37.54 ± 0.98	45.10 ± 1.43	10.23 ± 0.38	28.35 ± 0.65	23.05 ± 0.57
Monounsaturated FA	9.43 ± 0.18	4.26 ± 0.21	11.15 ± 0.43	27.82 ± 0.39	22.93 ± 0.44
Polyunsaturated FA	53.03 ± 2.07	50.64 ± 2.81	78.61 ± 3.27	43.83 ± 2.12	54.02 ± 1.82
LA: ALA	0.67 ± 0.07	0.76 ± 0.04	2.94 ± 0.11	9.06 ± 0.21	4.15 ± 0.18
Concentrates ingredients (% as fed)					
Maize grain				55.0	47.0
Triticale grain				10.0	10.0
Rapeseed meal				21.0	11.0
Sunflower meal				12.0	5.0
Hemp seed				-	25.0
Premix mineral and vitaminic				2.0	2.0

C—concentrates without hemp seed; Hs—concentrates with hemp seed. ** NEL: net energy lactation, was estimated in according NRC [25]. DM—dry matter; NDF—neutral detergent fiber; ADF—acid detergent fiber; FA—fatty acid; OA—oleic acid; LA—linoleic acid; ALA—α-linolenic acid.

**Table 3:** Effect of diet type (H = hay, SG = shrubs–grass mixture) and diet supplementation with hemp seed (Hs) on daily milk yield and composition (mean ± S.E.).

Item	Treatment				*p*-Value ^†^		
	Hay (H)		Shrubs–Grass (SG)				
	H-C	H-Hs	SG-C	SG-Hs	F	Hs	F × Hs
Milk yield (kg/d)							
Milk	1.464 ± 0.13 ^ab^	1.576 ± 0.18 ^a^	1.273 ± 0.17 ^c^	1.361 ± 0.11 ^b^	0.008	0.041	0.124
FPCM	1.397 ± 0.15 ^c^	1.721 ± 0.21 ^a^	1.316 ± 0.14 ^c^	1.579 ± 0.16 ^b^	0.038	<0.001	0.048
ECM	1.293 ± 0.23 ^b^	1.584 ± 0.15 ^a^	1.211 ± 0.13 ^b^	1.443 ± 0.21 ^ab^	0.011	0.004	0.320
Milk content							
Fat (%)	3.30 ± 0.15 ^c^	4.28 ± 0.12 ^ab^	3.92 ± 0.18 ^b^	4.88 ± 0.06 ^a^	<0.001	<0.001	0.513
Protein (%)	3.14 ± 0.07	3.21 ± 0.09	3.09 ± 0.12	3.04 ± 0.08	0.078	0.212	0.172
Lactose (%)	4.51 ± 0.02	4.47 ± 0.05	4.45 ± 0.02	4.39 ± 0.03	0.165	0.084	0.441
SNF (%)	8.33 ± 0.08	8.38 ± 0.11	8.22 ± 0.14	8.14 ± 0.09	0.112	0.093	0.276
Total solids (%)	11.63 ± 0.12 ^b^	12.66 ± 0.21 ^ab^	12.14 ± 0.10 ^ab^	13.03 ± 0.14 ^a^	0.043	0.028	0.195
MUL (mg/dL)	29.12 ± 0.19 ^b^	26.40 ± 0.16 ^c^	37.34 ± 0.22 ^a^	36.21 ± 0.20 ^a^	0.009	0.085	0.372
Cholesterol (mg/100 g)	15.43 ± 0.32 ^a^	12.84 ± 0.21 ^b^	10.21 ± 0.15 ^c^	8.92 ± 0.13 ^d^	0.008	0.019	0.085
Yield (g/d):							
Fat	48.31 ± 0.65 ^b^	67.45 ± 0.88 ^a^	49.90 ± 0.71 ^b^	66.42 ± 0.54 ^a^	0.102	<0.001	0.270
Protein	45.97 ± 0.55 ^b^	50.59 ± 0.74 ^a^	39.34 ± 0.48 ^c^	41.37 ± 0.40 ^c^	0.047	0.029	0.041
Lactose	66.03 ± 0.38 ^b^	70.45 ± 0.49 ^a^	56.65 ± 0.33 ^c^	59.75 ± 0.52 ^c^	0.039	0.090	0.075
Milk fat: protein	1.05 ± 0.05 ^c^	1.33 ± 0.04 ^b^	1.27 ± 0.07 ^b^	1.61 ± 0.07 ^a^	0.045	0.008	0.129

S.E.—standard error, ^†^ F—type of forage, Hs—hempseed, F × Hs. ^a,b,c^ = mean values within rows with different superscripts differ at *p* < 0.05. FPCM: fat and protein-corrected milk; ECM: energy-corrected milk; SNF: total solids non-fat; MUL: milk urea level.

**Table 4:** Effect of diet type (H = hay, SG = shrub–grass mixture) and diet supplementation with hemp seed (Hs) on milk fatty acids (FA) composition (% of total FA) (mean ± S.E.).

Item	Treatment				*p*-Value ^†^		
	Hay (H)		Shrubs–Grass (SG)				
	H-C	H-Hs	SG-C	SG-Hs	F	Hs	F × Hs
Total FA (g/100 g fat milk)	94.62 ± 1.05	94.59 ± 1.16	94.76 ± 1.32	94.43 ± 1.06	0.312	0.107	0.502
C4:0	1.87 ± 0.05 ^b^	2.03 ± 0.07 ^ab^	2.11 ± 0.05 ^ab^	2.46 ± 0.04 ^a^	0.023	0.271	0.232
C6:0	2.42 ± 0.09	2.49 ± 0.05	2.53 ± 0.11	2.35 ± 0.10	0.321	0.064	0.425
C8:0	2.55 ± 0.15	2.58 ± 0.09	2.48 ± 0.10	2.61 ± 0.12	0.126	0.297	0.572
C10:0	11.65 ± 0.41 ^a^	10.48 ± 0.36 ^ab^	9.07 ± 0.33 ^b^	7.88 ± 0.28 ^c^	0.014	0.029	0.279
C10:1 *cis*-9	0.22 ± 0.02 ^a^	0.18 ± 0.03 ^ab^	0.21 ± 0.02 ^a^	0.15 ± 0.02 ^b^	0.571	0.043	0.042
C12:0	4.72 ± 0.19 ^a^	3.73 ± 0.21 ^b^	4.34 ± 0.18 ^a^	3.41 ± 0.26 ^b^	0.302	0.003	0.488
C12:1 *cis*-9	0.07 ± 0.003	0.05 ± 0.005	0.08 ± 0.004	0.05 ± 0.002	0.129	0.038	0.234
C14:0	11.38 ± 0.36 ^a^	9.52 ± 0.33 ^b^	11.02 ± 0.44 ^a^	9.41 ± 0.31 ^b^	0.464	<0.001	0.453
C14:1 *cis*-9	0.19 ± 0.02 ^a^	0.12 ± 0.04 ^a^	0.14 ± 0.03 ^a^	0.07 ± 0.02 ^b^	0.012	0.002	0.039
C15:0	1.07 ± 0.04 ^a^	0.88 ± 0.03 ^ab^	0.92 ± 0.05 ^ab^	0.75 ± 0.03 ^b^	0.174	0.088	0.312
C15:1	0.39 ± 0.03	0.36 ± 0.02	0.37 ± 0.02	0.32 ± 0.02	0.141	0.635	0.299
C16:0	27.35 ± 1.07 ^a^	22.61 ± 1.39 ^b^	25.04 ± 1.22 ^ab^	20.82 ± 1.15 ^c^	0.005	0.008	0.434
C16:1 *cis*-9	0.88 ± 0.02 ^a^	0.52 ± 0.04 ^b^	0.58 ± 0.03 ^b^	0.47 ± 0.02 ^b^	0.003	0.030	0.017
C17:0	0.78 ± 0.04 ^a^	0.56 ± 0.03 ^ab^	0.55 ± 0.02 ^ab^	0.41 ± 0.02 ^b^	0.005	0.037	0.201
C17:1	0.19 ± 0.02	0.15 ± 0.01	0.26 ± 0.03	0.20 ± 0.02	0.016	0.057	0.398
C18:0	7.45 ± 0.33 ^b^	10.12 ± 0.54 ^a^	10.55 ± 0.35 ^a^	11.98 ± 0.39 ^a^	<0.001	0.006	0.018
C18:1 *trans*-(6 + 7 + 8)	0.10 ± 0.01 ^b^	0.18 ± 0.02 ^a^	0.12 ± 0.01 ^b^	0.23 ± 0.01 ^a^	0.053	0.035	0.172
C18:1 *trans*-9	0.23 ± 0.02 ^b^	0.26 ± 0.01 ^b^	0.30 ± 0.01 ^a^	0.37 ± 0.01 ^a^	0.082	0.064	0.502
C18:1 *trans*-10	0.18 ± 0.01 ^c^	0.35 ± 0.02 ^a^	0.26 ± 0.02 ^b^	0.41 ± 0.03 ^a^	0.165	0.006	0.457
C18:1 *trans*-11 (VA)	1.18 ± 0.11 ^c^	2.08 ± 0.09 ^b^	2.54 ± 0.15 ^b^	4.05 ± 0.21 ^a^	<0.001	<0.001	0.019
C18:1 *cis*-9 (OA)	18.13 ± 0.56 ^b^	21.34 ± 0.61 ^a^	18.65 ± 0.48 ^b^	20.01 ± 0.51 ^ab^	0.075	<0.001	0.037
C18:1 *cis*-(12 + 13)	0.27 ± 0.02 ^c^	0.35 ± 0.03 ^b^	0.41 ± 0.02 ^a^	0.48 ± 0.04 ^a^	0.024	0.039	0.348
C18:1 *trans* total	1.69 ± 0.12 ^c^	2.87 ± 0.19 ^b^	3.22 ± 0.18 ^b^	5.06 ± 0.22 ^a^	<0.001	<0.001	0.012
C18:1 *cis* total	18.40 ± 0.42 ^b^	21.69 ± 0.36 ^a^	19.06 ± 0.41 ^b^	20.49 ± 0.39 ^ab^	0.010	0.005	0.027
C18:1 total	20.09 ± 0.92 ^c^	24.56 ± 0.86 ^a^	22.28 ± 1.15 ^b^	25.55 ± 0.88 ^a^	0.003	<0.001	0.047
C18:2 *trans*-9, *trans*-12	0.21 ± 0.02	0.20 ± 0.04	0.22 ± 0.03	0.22 ± 0.02	0.515	0.367	0.187
C18:2 *cis*-9, *cis*-12 (LA)	2.71 ± 0.09 ^b^	3.42 ± 0.11 ^a^	3.48 ± 0.18 ^a^	3.95 ± 0.10 ^a^	0.012	0.033	0.223
CLA total	1.08 ± 0.04 ^c^	1.60 ± 0.07 ^b^	1.47 ± 0.05 ^b^	2.41 ± 0.09 ^a^	0.003	<0.001	0.091
CLA *cis*-9, *trans*-11 (RA)	0.97 ± 0.04 ^c^	1.48 ± 0.07 ^b^	1.37 ± 0.05 ^b^	2.29 ± 0.08 ^a^	0.007	<0.001	0.017
CLA *trans*-10, *cis*-12	0.11 ± 0.01	0.12 ± 0.02	0.10 ± 0.01	0.12 ± 0.01	0.083	0.131	0.040
C18:3 *cis*-6, *cis*-9, *cis*-12	0.05 ± 0.007	0.06 ± 0.008	0.07 ± 0.01	0.06 ± 0.009	0.232	0.091	0.118
C18:3 c-9, c-12, c-15 (ALA)	1.03 ± 0.08 ^c^	1.76 ± 0.11 ^b^	1.42 ± 0.09 ^b^	2.32 ± 0.13 ^a^	0.005	<0.001	0.013
C20:0	0.16 ± 0.03 ^c^	0.24 ± 0.02 ^b^	0.30 ± 0.02 ^ab^	0.39 ± 0.02 ^a^	0.009	0.007	0.161
C20:4 n-6 (AA)	0.23 ± 0.02	0.22 ± 0.03	0.26 ± 0.02	0.24 ± 0.02	0.211	0.364	0.499
C20:5 n-3 (EPA)	0.05 ± 0.006 ^c^	0.11 ± 0.01 ^b^	0.12 ± 0.01 ^b^	0.20 ± 0.02 ^a^	0.004	0.008	0.271
C22:0	0.02 ± 0.002	0.03 ± 0.001	0.02 ± 0.001	0.02 ± 0.001	0.247	0.158	0.429
C22:5 n-3 (DPA)	0.09 ± 0.001 ^c^	0.20 ± 0.002 ^b^	0.16 ± 0.002 ^b^	0.28 ± 0.002 ^a^	0.036	0.007	0.288
Unidentified fatty acids	1.09 ± 0.03	1.22 ± 0.04	0.98 ± 0.03	1.02 ± 0.03	0.217	0.112	0.324

S.E.—standard error, ^†^ F—type of forage, Hs—hempseed, F × Hs. ^a,b,c^ = mean values within rows with different superscripts differ at *p* < 0.05. VA: vaccenic acid; OA: oleic acid; LA: linoleic acid; CLA: conjugated linoleic acid; RA: rumenic acid; ALA: α-linolenic acid; AA: arachidonic acid; EPA: eicosapentaenoic acid; DPA: docosapentaenoic acid.

**Table 5:** Effect of diet type (H = hay, SG = shrubs–grass mixture) and diet supplementation with hemp seed (Hs) on milk fatty acid sums (% of total FA) (mean ± S.E.).

Item	Treatment				*p*-Value ^†^		
	Hay (H)		Shrubs–Grass (SG)				
	H-C	H-Hs	SG-C	SG-Hs	F	Hs	F × Hs
Ʃ SFA	71.42 ± 1.78 ^a^	65.27 ± 1.23 ^b^	68.93 ± 1.19 ^ab^	62.49 ± 1.08 ^c^	0.006	<0.001	0.527
Ʃ MUFA	22.03 ± 1.13 ^b^	25.94 ± 1.25 ^a^	22.89 ± 1.41 ^b^	26.81 ± 1.07 ^a^	0.357	0.002	0.139
Ʃ *trans* total	2.48 ± 0.09 ^c^	3.58 ± 0.12 ^b^	4.07 ± 0.10 ^b^	5.80 ± 0.14 ^a^	0.007	0.032	0.016
Ʃ *trans* MUFA	2.27 ± 0.11 ^c^	3.38 ± 0.07 ^b^	3.85 ± 0.12 ^b^	5.58 ± 0.10 ^a^	0.005	0.009	0.218
Ʃ *cis* MUFA	19.76 ± 0.85 ^b^	22.56 ± 1.15 ^a^	20.07 ± 0.90 ^b^	21.23 ± 1.07 ^ab^	0.063	0.004	0.014
Ʃ PUFA	5.45 ± 0.21 ^c^	7.57 ± 0.33 ^b^	7.20 ± 0.27 ^b^	9.67 ± 0.41 ^a^	0.007	<0.001	0.272
Ʃ n-6 PUFA ^1^	2.99 ± 0.10 ^b^	3.70 ± 0.14 ^ab^	3.81 ± 0.15 ^ab^	4.25 ± 0.10 ^a^	0.033	0.011	0.163
Ʃ n-3 PUFA ^2^	1.17 ± 0.08 ^c^	2.07 ± 0.10 ^ab^	1.70 ± 0.09 ^b^	2.81 ± 0.12 ^a^	0.005	<0.001	0.295
Ʃ UFA	27.48 ± 1.08 ^c^	33.51 ± 1.29 ^ab^	30.09 ± 1.14 ^b^	36.48 ± 1.57 ^a^	0.002	<0.001	0.063
Ʃ *trans* FA + SFA	73.90 ± 1.78 ^a^	68.85 ± 1.16 ^b^	73.00 ± 1.27 ^a^	68.29 ± 1.06 ^b^	0.103	<0.001	0.186
Ʃ *cis* FA ^3^	23.92 ± 1.25 ^b^	28.42 ± 1.07 ^a^	25.58 ± 1.52 ^ab^	28.28 ± 1.18 ^a^	0.027	<0.001	0.031
HFA	43.45 ± 0.97 ^a^	35.86 ± 1.13 ^c^	40.40 ± 1.07 ^b^	33.64 ± 0.88 ^c^	0.012	<0.001	0.055
hFA	23.85 ± 1.18 ^c^	29.26 ± 1.31 ^a^	26.26 ± 1.11 ^b^	30.16 ± 1.14 ^a^	0.003	<0.001	0.021
Product/substrate ratios (∆9 desaturase activity):							
∆9C14	0.016 ± 0.002 ^a^	0.012 ± 0.001 ^a^	0.012 ± 0.004 ^a^	0.007 ± 0.002 ^b^	0.087	0.006	0.042
∆9C16	0.031 ± 0.004 ^a^	0.022 ± 0.007 ^b^	0.023 ± 0.005 ^b^	0.022 ± 0.004 ^b^	0.262	0.028	0.044
∆9C18	0.709 ± 0.04 ^a^	0.678 ± 0.03 ^a^	0.638 ± 0.02 ^b^	0.625 ± 0.03 ^b^	0.014	0.152	0.309
RA/RA + VA	0.451 ± 0.03 ^a^	0.416 ± 0.07 ^a^	0.350 ± 0.02 ^b^	0.361 ± 0.05 ^b^	0.175	0.091	0.152
∆^9^—desaturase index (DI)	0.299 ± 0.01	0.346 ± 0.01	0.297 ± 0.03	0.331 ± 0.02	0.166	0.014	0.297

S.E.—standard error, ^†^ F—type of forage, Hs—hempseed, F × Hs. ^a,b,c^ = mean values within rows with different superscripts differ at *p* < 0.05. HFA: hypercholesterolemic FA (C12:0 + C14:0 + C16:0); hFA: hypocholesterolemic FA (*cis*-C18:1 + PUFA); ^1^ Sum of 18:2 *cis*-9, *cis*-12; 18:3 *cis*-6, *cis*-9, *cis*-12; 20:4 n-6, and 22:6 n-6. ^2^ Sum of 18:3 *cis*-9, *cis*-12, *cis*-15; 20:5 n-3 (EPA); 22:5 n-3 (DPA); ^3^ Sum of *cis*-MUFA and *cis*-PUFA.

**Table 6:** Effect of diet type (H = hay, SG = shrubs–grass mixture) and diet supplementation with hemp seed (Hs) on goat milk health and risk indices (mean ± S.E.).

Item	Treatment				*p*-Value ^†^		
	Hay (H)		Shrubs–Grass (SG)				
	H-C	H-Hs	SG-C	SG-Hs	F	Hs	F × Hs
PUFA/SFA	0.076 ± 0.009 ^b^	0.116 ± 0.017 ^ab^	0.104 ± 0.011 ^ab^	0.155 ± 0.008 ^a^	<0.001	<0.001	0.067
HFA/UFA	1.581 ± 0.071 ^a^	1.070 ± 0.064 ^c^	1.343 ± 0.087 ^b^	0.922 ± 0.107 ^c^	0.043	<0.001	0.521
n-6/n-3 FA	2.55 ± 0.092 ^a^	1.79 ± 0.071 ^b^	2.24 ± 0.107 ^a^	1.51 ± 0.057 ^b^	0.023	<0.001	0.136
AI	2.82 ± 0.123 ^a^	1.92 ± 0.094 ^b^	2.44 ± 0.141 ^a^	1.70 ± 0.086 ^b^	0.018	<0.001	0.072
TI	2.41 ± 0.174 ^a^	1.69 ± 0.110 ^b^	2.11 ± 0.167 ^a^	1.01 ± 0.073 ^c^	0.039	<0.001	0.033
h/H FA	0.549 ± 0.035 ^c^	0.816 ± 0.046 ^a^	0.650 ± 0.071 ^b^	0.897 ± 0.055 ^a^	0.041	<0.001	0.264
LA/ALA	2.63 ± 0.394 ^a^	1.94 ± 0.253 ^b^	2.45 ± 0.302 ^a^	1.70 ± 0.178 ^b^	0.085	0.007	0.138
OA/PA	0.663 ± 0.173 ^b^	0.944 ± 0.091 ^a^	0.745 ± 0.107 ^b^	0.961 ± 0.092 ^a^	0.028	<0.001	0.025
HPI	0.338 ± 0.027 ^b^	0.492 ± 0.020 ^a^	0.387 ± 0.031 ^b^	0.547 ± 0.028 ^a^	0.012	0.006	0.224
NVI	1.01 ± 0.093 ^c^	1.53 ± 0.062 ^ab^	1.31 ± 0.071 ^b^	1.80 ± 0.082 ^a^	0.040	0.002	0.321
PI	4.77 ± 0.740 ^c^	6.94 ± 0.861 ^b^	6.32 ± 0.728 ^b^	8.59 ± 0.511 ^a^	<0.001	0.036	0.209
DFA	34.93 ± 2.107 ^c^	43.63 ± 1.676 ^ab^	40.64 ± 2.219 ^b^	48.46 ± 1.875 ^a^	0.008	<0.001	0.063

S.E.—standard error, ^†^ F—type of forage, Hs—hempseed, F × Hs. ^a,b,c^ = mean values within rows with different superscripts differ at *p* < 0.05.PUFA: polyunsaturated FA; SFA: saturated FA; HFA: hypercholesterolaemic FA; UFA: unsaturated fatty acid; AI: atherogenicity index; TI: thrombogenicity index; h/H (hypocholesterolemic/hypercholesterolemic ratio); LA: linoleic acid (C18:2 n-6); ALA: α-linolenic acid (C18:3 n-3); OA: oleic acid (C18:1 c9); PA: palmitic acid (C16:0); HPI: health-promoting index; NVI: nutritive value indices; PI: polyunsaturated index; DFA: desirable FA.

**Table 7:** Levels of fat-soluble vitamins and total antioxidant capacity (TAC) in milk (raw: R, pasteurized: P and stored: S) from goats (mean ± S.E.).

Item		Treatment				*p*-Value ^†^		
		Hay (H)		Shrubs–Grass (SG)				
		H-C	H-Hs	SG-C	SG-Hs	F	Hs	F × Hs
α-tocopherol (µg/g)	R	0.87 ± 0.131 ^cA^	1.36 ± 0.247 ^bA^	1.73 ± 0.103 ^abA^	2.45 ± 0.159 ^aA^	0.005	0.008	0.151
	S	0.65 ± 0.114 ^cB^	0.94 ± 0.231 ^bB^	1.36 ± 0.259 ^abB^	1.88 ± 0.186 ^aB^	0.006	0.004	0.089
Retinol (µg/g)	R	0.58 ± 0.072 ^bA^	0.63 ± 0.095 ^bA^	0.86 ± 0.104 ^aA^	0.85 ± 0.070 ^aA^	0.009	0.257	0.385
	S	0.45 ± 0.055 ^bB^	0.50 ± 0.078 ^bB^	0.72 ± 0.091 ^aB^	0.70 ± 0.086 ^aB^	0.003	0.077	0.218
TAC (µM TE/mL)	R	3.47 ± 0.304 ^bA^	3.91 ± 0.462 ^bA^	3.72 ± 0.351 ^bA^	4.28 ± 0.256 ^aA^	0.024	0.008	0.044
	P	3.39 ± 0.296 ^bA^	3.78 ± 0.266 ^bA^	3.68 ± 0.295 ^bA^	4.07 ± 0.452 ^aA^	0.037	0.041	0.307
	S	2.62 ± 0.291 ^bB^	3.05 ± 0.174 ^aB^	3.16 ± 0.247 ^aB^	3.41 ± 0.311 ^aB^	0.007	0.029	0.077

S.E.—standard error, ^†^ F—type of forage, Hs—hempseed, F × Hs. ^a,b,c^ = mean values within rows with different superscripts differ at *p* < 0.05. ^A,B^ = Mean values within a column without a common superscript corresponding to a parameter (R vs. S) differ significantly (*p* < 0.05).

**Table 8:** Pearson correlation coefficients between total antioxidant capacity (TAC, µM TE/mL), concentrations of major FA (% of total FA) in milk and fat-soluble vitamins content (µg/g) in milk in goats fed hay-based diets or mixed shrubs–grass rangeland, supplemented or not with hemp seeds.

	TAC	C18:1 total	C18:1 c-9	C18: t-11	C18:2 n-6	C18:3 n-3	CLA c-9 t-11	Retinol
C18:1 total	−0.151							
C18:1 c-9 (OA)	0.055	0.521 ***						
C18: t-11 (VA)	0.009	0.695 ***	0.233 *					
C18:2 n-6 (LA)	−0.380 **	0.612 ***	0.273 *	0.837 ***				
C18:3 n-3 (ALA)	−0.528 ***	0.759 ***	0.389 **	0.883 ***	0.755 ***			
CLA c-9 t-11 (RA)	−0.015	0.714 ***	0.412 **	0.923 ***	0.780 ***	0.906 ***		
retinol	0.358 **	0.369 **	0.002	0.683 ***	0.584 ***	0.474 **	0.555 ***	
α-tocopherol	0.615 ***	0.596 ***	0.253 *	0.948 ***	0.859 ***	0.815 ***	0.888 ***	0.736 ***

* *p* < 0.05; ** *p* < 0.01 *** *p* < 0.001.

## Data Availability

Data are available from the first author upon request.
